# Complete genome sequence of *Desulfohalobium retbaense* type strain (HR_100_^T^)

**DOI:** 10.4056/sigs.581048

**Published:** 2010-01-28

**Authors:** Stefan Spring, Matt Nolan, Alla Lapidus, Tijana Glavina Del Rio, Alex Copeland, Hope Tice, Jan-Fang Cheng, Susan Lucas, Miriam Land, Feng Chen, David Bruce, Lynne Goodwin, Sam Pitluck, Natalia Ivanova, Konstantinos Mavromatis, Natalia Mikhailova, Amrita Pati, Amy Chen, Krishna Palaniappan, Loren Hauser, Yun-Juan Chang, Cynthia D. Jeffries, Christine Munk, Hajnalka Kiss, Patrick Chain, Cliff Han, Thomas Brettin, John C. Detter, Esther Schüler, Markus Göker, Manfred Rohde, Jim Bristow, Jonathan A. Eisen, Victor Markowitz, Philip Hugenholtz, Nikos C. Kyrpides, Hans-Peter Klenk

**Affiliations:** 1 DSMZ – German Collection of Microorganisms and Cell Cultures GmbH, Braunschweig,; Germany; 2 DOE Joint Genome Institute, Walnut Creek, California, USA; 3 Los Alamos National Laboratory, Bioscience Division, Los Alamos, New Mexico, USA; 4 Lawrence Livermore National Laboratory, Livermore, California, USA; 5 Oak Ridge National Laboratory, Oak Ridge, Tennessee, USA; 6 University of California Davis Genome Center, Davis, California, USA; 7 HZI – Helmholtz Centre for Infection Research, Braunschweig, Germany; 8 Biological Data Management and Technology Center, Lawrence Berkeley National Laboratory, Berkeley, California, USA

**Keywords:** sulfate-reducer, Gram-negative, mesophile, moderately halophilic, strictly anaerobic, hydrogen utilization, hypersaline lake, *Desulfohalobiaceae*, *Deltaproteobacteria*, *Proteobacteria*, GEBA

## Abstract

*Desulfohalobium retbaense* (Ollivier *et al*. 1991) is the type species of the polyphyletic genus *Desulfohalobium*, which comprises, at the time of writing, two species and represents the family *Desulfohalobiaceae* within the *Deltaproteobacteria*. *D. retbaense* is a moderately halophilic sulfate-reducing bacterium, which can utilize H_2_ and a limited range of organic substrates, which are incompletely oxidized to acetate and CO_2_, for growth. The type strain HR_100_^T^ was isolated from sediments of the hypersaline Retba Lake in Senegal. Here we describe the features of this organism, together with the complete genome sequence and annotation. This is the first completed genome sequence of a member of the family *Desulfohalobiaceae*. The 2,909,567 bp genome (one chromosome and a 45,263 bp plasmid) with its 2,552 protein-coding and 57 RNA genes is a part of the *** G****enomic* *** E****ncyclopedia of* *** B****acteria and* *** A****rchaea * project.

## Introduction

Strain HR_100_^T^ (= DSM 5692) is the type strain of the species *Desulfohalobium retbaense* [1]. HR_100_^T^ is the only strain available from culture collections belonging to this species and was isolated from surface sediments of the hypersaline Retba Lake in Senegal (Western Africa). This strain was the first cultivated sulfate-reducing bacterium, which grows in media containing NaCl concentrations up to 24% and the first described hydrogenotrophic anaerobe able to grow at salinities above 10% [1]. Interestingly, the total salt concentration of the Retba Lake was 34% at the time of sampling, which would indicate that cells of this strain were not able to proliferate in the habitat from which they were originally isolated. This phenomenon was later also reported in a study on the diversity of sulfate-reducing bacteria in hypersaline sediments of the Great Salt Lake (Utah) [2]. This effect could either be explained by niches of lower salinity in the respective habitats, which would allow proliferation at distinct sites or, alternatively, that the *in vitro* halotolerance of these strains is different from the salt tolerance in the natural environment. One reason for the observed growth inhibition of sulfate-reducers at salinities above 24% may be the energy expensive synthesis of compatible osmotic solutes, which are required in large amounts to retain cellular integrity at high external salt concentrations. Under anoxic conditions bacteria that depend on sulfate as electron acceptor gain less energy than microorganisms that use photosynthesis or denitrification for growth, so that the latter metabolic types have a selective advantage in hypersaline environments [3]. Here we present a summary classification and a set of features for *D. retbaense* strain HR_100_^T^, together with the description of the complete genomic sequencing and annotation.

## Classification and features

So far, no 16S rRNA gene sequences with high similarity (>95%) to the sequence of *D. retbaense* have been deposited in public databases, although several anoxic sediments with high salinity have been analyzed by cultivation independent methods (as of October 2009) since *D. retbaense* was described. Consequently, it appears that cells of sulfate-reducing bacteria related to this species are of very low abundance in most hypersaline environments. Besides several strains of the genus *Desulfovibrio*, the only other member of the order *Desulfovibrionales* with a sequenced genome is *Desulfomicrobium baculatum* type strain X^T^ [4].

**Figure 1:**
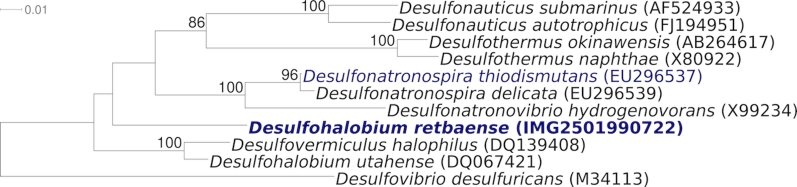
Phylogenetic tree highlighting the position of strain HR_100_^T^, *D. retbaense* DSM 5692, relative to the other type strains within the family. The tree was inferred from 1,386 aligned characters [6,7] of the 16S rRNA gene sequence under the maximum likelihood criterion [8] and rooted in accordance with the type strain of the order *Desulfovibrionales*. The branches are scaled in terms of the expected number of substitutions per site. Numbers above branches are support values from 1,000 bootstrap replicates if larger than 60%. Lineages with type strain genome sequencing projects registered in GOLD [9] are shown in blue, published genomes in bold.

*D. retbaense* is the type species of the genus *Desulfohalobium*, which represents the recently proposed family *Desulfohalobiaceae* within the class *Deltaproteobacteria* [5]. The genus *Desulfohalobium* is currently polyphyletic due to the species *D. utahense*, which is phylogenetically more closely related to *Desulfovermiculus halophilus*, with high bootstrapping support in the 16S rRNA tree (Figure 1) Also, both share a 16S rRNA gene sequence similarity of 96.9%, whereas the two *Desulfohalobium* species display a sequence similarity of only 90.5%. Hence,**it is possible that the species *D. utahense* has been misclassified, although it**appears to be phenotypically more similar to *D. retbaense* than to *Desulfovermiculus halophilus* [10]. The taxonomy of the two genera thus needs to be reconsidered.

Figure 1 shows the phylogenetic neighborhood of *D. retbaense* strain HR_100_^T^ in a 16S rRNA based tree. The two 16S rRNA gene copies in the genome of strain HR_100_^T^ do not differ from each other, and differ by four nucleotides from the previously published 16S rRNA sequence generated from DSM 5692 (X99235). The difference between the genome data and the reported 16S rRNA gene sequence is most likely due to sequencing errors in the previously reported sequence data.

**Figure 2:**
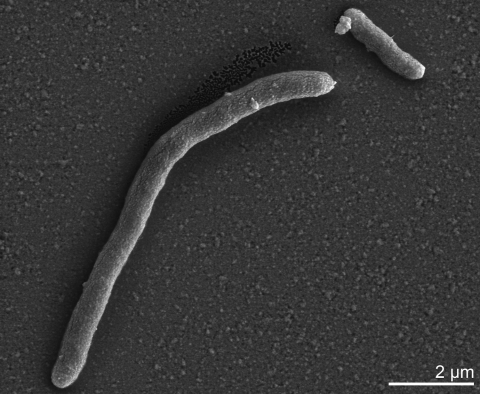
Scanning electron micrograph of cells of *D. retbaense* strain HR_100_^T^

Cells of *D. retbaense* HR_100_^T^ are straight to slightly curved rods with rounded ends (Table 1 and Figure 2). They have dimensions of 0.7-0.9 x 1-3 µm and stain Gram-negative. In medium containing lactate as substrate, cells can form filaments up to 20 µm in length. Motility is conferred by one or two polar flagella [1].

**Table 1:** Classification and general features of *D. retbaense* strain HR_100_^T^ in accordance with the MIGS recommendations [11]

**MIGS ID**	**Property**	**Term**	**Evidence code**	
	Current classification	Domain *Bacteria*		TAS [12]
		Phylum *Proteobacteria*		TAS [13]
		Class *Deltaproteobacteria*		TAS [14,15]
		Order *Desulfovibrionales*		TAS [14]
		Family *Desulfohalobiaceae*		TAS [14]
		Genus *Desulfohalobium*		TAS [1]
		Species *Desulfohalobium retbaense*		TAS [1]
		Type strain HR_100_		TAS [1]
	Gram stain	negative		TAS [1]
	Cell shape	rod with rounded ends		TAS [1]
	Motility	motile (one or two polar flagella)		TAS [1]
	Sporulation	nonsporulating		TAS [1]
	Temperature range	25-43°C		TAS [1]
	Optimum temperature	37-40°C		TAS [1]
	Salinity	>0-240 g/l (optimum 100 g/l)		TAS [1]
MIGS-22	Oxygen requirement	obligate anaerobic		TAS [1]
	Carbon source	acetate, biotrypcase, yeast extract		TAS [1]
	Energy source	H_2_, formate, lactate, ethanol, pyruvate		TAS [1]
MIGS-6	Habitat	hypersaline sediments		TAS [1]
MIGS-15	Biotic relationship	free living		NAS
MIGS-14	Pathogenicity	none		TAS [16]
	Biosafety level	1		TAS [16]
	Isolation	surface sediment		TAS [1]
MIGS-4	Geographic location	Retba Lake, Senegal		TAS [1]
MIGS-5	Sample collection time	1989		NAS
MIGS-4.1 MIGS-4.2	Latitude, Longitude	14.84, -17.23		NAS
MIGS-4.3	Depth	not reported		
MIGS-4.4	Altitude	-4 m		TAS [1]

Evidence codes - IDA: Inferred from Direct Assay (first time in publication); TAS: Traceable Author Statement (i.e., a direct report exists in the literature); NAS: Non-traceable Author Statement (i.e., not directly observed for the living, isolated sample, but based on a generally accepted property for the species, or anecdotal evidence). These evidence codes are from the Gene Ontology project [17]. If the evidence code is IDA, then the property was directly observed for a live isolate by one of the authors or an expert mentioned in the acknowledgments.

Strain HR_100_^T^ is halophilic and requires NaCl and MgCl_2_ for growth. The optimal NaCl concentration for growth is near 10% and salinities up to 24% are tolerated. The pH range for growth is 5.5 to 8.0 with an optimum between pH 6.5 and 7.0. Growth of this strain occurs at temperatures from 25 to 43°C and is optimal between 37 and 40°C [1].

The nutritional characteristics of strain HR_100_^T^ are as follows: Vitamins and an organic carbon source are required for growth in mineral medium. Hydrogen is utilized mixotrophically with acetate, yeast extract or biotrypcase as the carbon source, but not autotrophically. Organic carbon sources supporting growth are formate, ethanol, pyruvate and lactate. Sulfate, sulfur, thiosulfate and sulfite are used as electron acceptors and are reduced to H_2_S. In the absence of sulfate pyruvate can be also utilized fermentatively [1].

### Chemotaxonomy

Spectrophotometry of cell extracts indicate the presence of soluble *c*-type cytochromes having absorption maxima at 418.5, 522.5 and 552 nm in the reduced state, which would be characteristic for cytochrome *c*_3_. A dissimilatory sulfite reductase with a similar absorption spectrum as the enzyme of *Desulfomicrobium baculatum* (desulforubidin) was detected, but no desulfoviridin, which is diagnostic for members of the genus *Desulfovibrio* [1]. The respiratory lipoquinone composition of strain HR_100_^T^ has not been reported, but the moderately related species *Desulfovermiculus halophilus* was shown to contain the menaquinone MK-7 [18]. The whole cell fatty acid pattern of strain HR_100_^T^ is dominated by straight- and branched-chain saturated fatty acids (approx. 68%). Branched chain saturated fatty acids account for 30% of the total fatty acids, with iso-C_15:0_ predominating. In addition, the fatty acid profile contains branched-chain, mono-unsaturated fatty acids, such as iso-C_17:1ω7c_ and branched C_18:1 ω 6_ [1].

## Genome sequencing and annotation

### Genome project history

This organism was selected for sequencing on the basis of its phylogenetic position, and is part of the *** G****enomic* *** E****ncyclopedia of* *** B****acteria and* *** A****rchaea * project [19]. The genome project is deposited in the Genomes OnLine Database [9] and the complete genome sequence is available in GenBank. Sequencing, finishing and annotation were performed by the DOE Joint Genome Institute (JGI). A summary of the project information is shown in Table 2.

**Table 2:** Genome sequencing project information

**MIGS ID**	**Property**	**Term**
MIGS-31	Finishing quality	Finished
MIGS-28	Libraries used	Two genomic libraries - 8 kb pMCL200 and fosmid pcc1Fos
MIGS-29	Sequencing platforms	ABI3730
MIGS-31.2	Sequencing coverage	10.7× Sanger
MIGS-30	Assemblers	phrap
MIGS-32	Gene calling method	Prodigal
	INSDC ID	CP001734 (chromosome) CP001735 (plasmid)
	GenBank Date of Release	2009/09/14
	GOLD ID	Gc01111
	NCBI project ID	29199
	Database: IMG-GEBA	2501939614
MIGS-13	Source material identifier	DSM 5692
	Project relevance	Tree of Life, GEBA

### Growth conditions and DNA isolation

*D. retbaense* strain HR_100_^T^, DSM 5692, was grown anaerobically in DSMZ medium 499 [20] at 35°C. DNA was isolated from 1-1.5 g of cell paste using Qiagen Genomic 500 DNA Kit (Qiagen, Hilden, Germany) following the manufacturer's instructions.

### Genome sequencing and assembly

The genome was sequenced using a combination of 8 kb and fosmid DNA libraries. All general aspects of library construction and sequencing performed at the JGI can be found at the http://www.jgi.doe.gov/. The Phred/Phrap/Consed software package (http://www.phrap.com) was used for sequence assembly and quality assessment. Possible mis-assemblies were corrected with Dupfinisher [21] or transposon bombing of bridging clones [22]. Gaps between contigs were closed by editing in Consed, custom primer walk or PCR amplification. Sanger finishing reads (n=889) were produced to close gaps and to raise the quality of the finished sequence. The error rate of the completed genome sequence is less than 1 in 100,000. The final assembly consists of 42,114 Sanger reads. Together all sequence provided 10.7× coverage of the genome.

### Genome annotation

Genes were identified using Prodigal [23] as part of the Oak Ridge National Laboratory genome annotation pipeline, followed by a round of manual curation using the JGI GenePRIMP pipeline [24]. The predicted CDSs were translated and used to search the National Center for Biotechnology Information (NCBI) nonredundant database, UniProt, TIGRFam, Pfam, PRIAM, KEGG, COG, and InterPro databases. Additional gene prediction analysis and manual functional annotation was performed within the Integrated Microbial Genomes Expert Review (IMG-ER) platform [25].

## Genome properties

**Figure 3:**
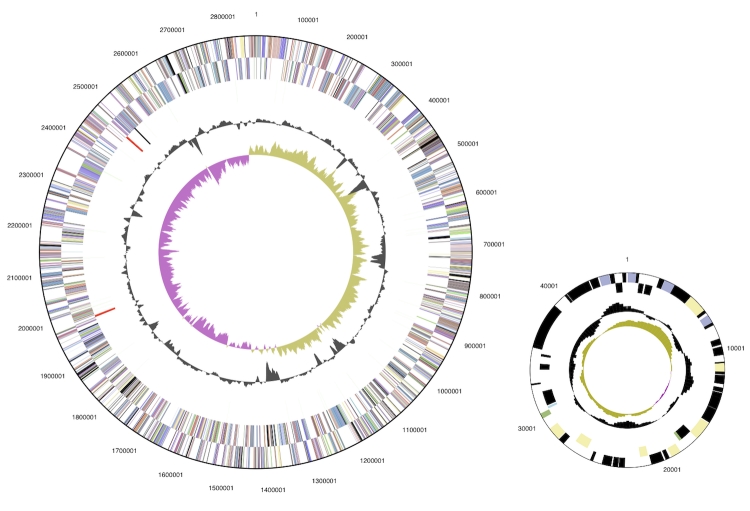
Graphical circular map of the genome. From outside to the center: Genes on forward strand (color by COG categories), Genes on reverse strand (color by COG categories), RNA genes (tRNAs green, rRNAs red, other RNAs black), GC content, GC skew.

The 2,909,567 bp genome consists of a 2,864,304 bp long chromosome and a 45,263 bp long plasmid with a 57.3% GC content (Table 3 and Figure 3). Of the 2,609 genes predicted, 2,552 were protein coding genes, and 57 RNAs; 29 pseudogenes were also identified. The majority of the protein-coding genes (73.6%) were assigned with a putative function while those remaining were annotated as hypothetical proteins. The distribution of genes into COGs functional categories is presented in Table 4.

**Table 3:** Genome Statistics

**Attribute**	Value	% of Total
Genome size (bp)	2,909,567	100.00%
DNA coding region (bp)	2,510,084	86.27%
DNA G+C content (bp)	1,666,078	57.33%
Number of replicons	2	
Extrachromosomal elements	1	
Total genes	2,609	100.00%
RNA genes	57	2.18%
rRNA operons	2	
Protein-coding genes	2,552	97.82%
Pseudo genes	29	1.11%
Genes with function prediction	1,920	73.59%
Genes in paralog clusters	266	10.20%
Genes assigned to COGs	1,976	75.74%
Genes assigned Pfam domains	1,968	75.43%
Genes with signal peptides	456	17.48%
Genes with transmembrane helices	634	24.30%
CRISPR repeats	1	

**Table 4:** Number of genes associated with the general COG functional categories

**Code**	**value**	**%age**	**Description**
J	151	5.9	Translation, ribosomal structure and biogenesis
A	0	0.0	RNA processing and modification
K	94	3.7	Transcription
L	129	5.1	Replication, recombination and repair
B	2	0.1	Chromatin structure and dynamics
D	28	1.1	Cell cycle control, mitosis and meiosis
Y	0	0.0	Nuclear structure
V	21	0.8	Defense mechanisms
T	168	6.6	Signal transduction mechanisms
M	146	5.7	Cell wall/membrane biogenesis
N	74	2.9	Cell motility
Z	0	0.0	Cytoskeleton
W	0	0.0	Extracellular structures
U	82	3.2	Intracellular trafficking and secretion
O	100	3.9	Posttranslational modification, protein turnover, chaperones
C	191	7.5	Energy production and conversion
G	101	4.0	Carbohydrate transport and metabolism
E	188	7.4	Amino acid transport and metabolism
F	53	2.1	Nucleotide transport and metabolism
H	109	4.3	Coenzyme transport and metabolism
I	43	1.7	Lipid transport and metabolism
P	95	3.7	Inorganic ion transport and metabolism
Q	20	0.8	Secondary metabolites biosynthesis, transport and catabolism
R	212	8.3	General function prediction only
S	152	6.0	Function unknown
-	633	24.8	Not in COGs

## Insights from the genome sequence

### Electron donor utilization

**Figure 4:**
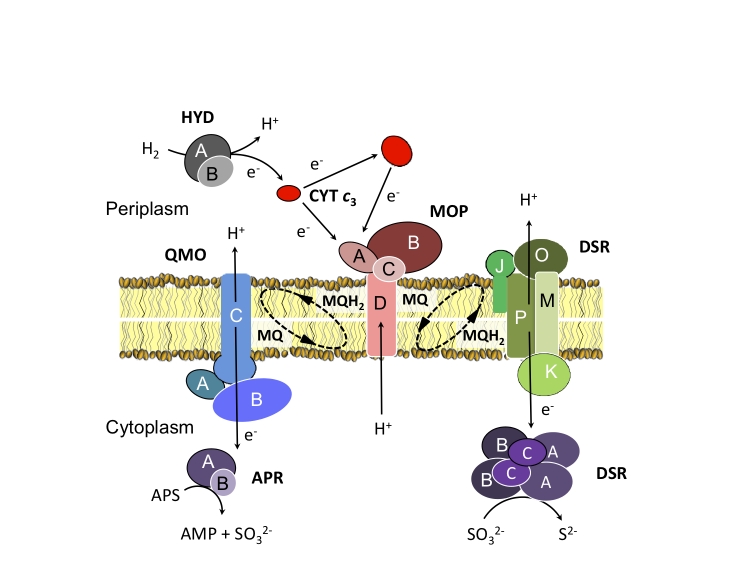
Proposed organization of the electron transfer chain in *D. retbaense* with H_2_ as electron donor and sulfate as electron acceptor. Gene products are designated according to the information given in Supplementary Table 1. Subunits of multiprotein complexes are labeled with capital letters. Abbreviations: APS, adenosine-5'-phosphosulfate; AMP, adenosine monophosphate; MQ, menaquinone; MQH2, dihydromenaquinone.

Similar to representatives of the genus *Desulfovibrio,* the preferred substrates of *D. retbaense* are H_2_ and lactate, the latter which is incompletely oxidized to acetate. Several genes could be identified that are involved in H_2_ dependent sulfate reduction in *Desulfovibrio* species. It is assumed that in species of this genus H_2_ is oxidized by periplasmic Fe- or NiFeSe-hydrogenases and the resulting electrons are transferred to a pool of periplasmic cytochrome *c*_3_. Then, membrane-bound protein complexes transfer electrons from the pool of reduced cytochrome *c*_3_ to menaquinone or directly to cytoplasmic enzymes involved in the reduction of sulfate to sulfide [26]. Recently, a novel molybdopterin oxidoreductase (Mop) could be identified in *Desulfovibrio* desulfuricans G20 that may represent a periplasm-facing transmembrane complex, which shuttles electrons from cytochrome *c*_3_ to the menaquinone pool [27]. It is thought that electrons are transferred from the reduced quinone pool to adenosine phosphosulfate and sulfite via the membrane-bound respiratory complexes Qmo [28] and Dsr [29], respectively. A similar electron transfer chain for the oxidation of H_2_ with sulfate appears to be functional in *D. retbaense*: The uptake of H_2_ is probably catalyzed in this species by a heterodimeric NiFe- or NiFeSe-hydrogenase encoded by the genes Dret_0265 (*hydB*) and Dret_0266 (*hydA*). Six genes of the completed genome were annotated as cytochromes class III containing at least one domain with homology to a tetraheme cytochrome *c*_3_. Electrons could be transferred from the reduced cytochrome c pool to menaquinone by a putative Mop complex (Dret_0270/Dret_0273) that is located in close proximity to the hydrogenase genes. A membrane-bound Qmo complex (Dret_1963, Dret_1964, and Dret_1965) was also identified adjacent to the genes of the dissimilatory adenylyl sulfate reductase (*aprAB*). Likewise, genes encoding the five subunits DsrMKJOP complex (Dret_0235/Dret_0239) were found close to genes of the α and β subunits of the dissimilatory sulfite reductase (*dsrAB*). Hence, it appears that the proposed organization of the electron transfer chain from H_2_ to sulfate seems to be conserved not only in species of the genus *Desulfovibrio*, but also in other sulfate-reducing members of the *Deltaproteobacteria*. In Figure 4 an illustration of the hypothetical electron transfer chain in *D. retbaense* is given, which is mainly based on results previously obtained with H_2_-utilizing *Desulfovibrio* species.

Several genes that are involved in the incomplete oxidation of lactate to acetate could be detected in the complete genome sequence. The transport of lactate in the cytoplasm is probably facilitated by a specific permease encoded by the gene Dret_1039. Following transport, lactate is oxidized to pyruvate by a putative L-lactate dehydrogenase (Dret_0157). Pyruvate is then oxidatively decarboxylated by a pyruvate ferredoxin oxidoreductase to acetyl-CoA. Interestingly, the gene Dret_1036 encoding a homodimeric pyruvate ferredoxin oxidoreductase is located in close proximity to the lactate permease gene and genes responsible for the substrate level phosphorylation of ADP to ATP via conversion of acetyl-CoA to acetate, i.e. phosphotransacetylase (Dret_1035) and acetate kinase (Dret_1034).

Besides substrate level phosphorylation, generation of ATP is also possible by the utilization of a chemiosmotic proton gradient through a F0F1 ATP synthase complex, which is encoded at two different sites of the genome. One gene cluster encodes the cytoplasmic F1 part along with the B-subunit of the membrane-bound F0 complex (Dret_2211/Dret_2217), whereas the remaining F0 subunits A (Dret_2087) and C (Dret_2086) are encoded elsewhere.

### Intermediary carbon metabolism

*D. retbaense* is not able to grow autotrophically with CO_2_ as carbon source and needs acetate or complex carbon sources for growth with H_2_ as energy source. Intermediary carbon compounds required as precursors for the biosynthesis of cellular components are probably synthesized by a partial reverse tricarboxylic acid (TCA) cycle. A possible pathway for the assimilation of acetate starts with the synthesis of pyruvate from acetyl-CoA through a carboxylating reaction catalyzed by the pyruvate ferredoxin oxidoreductase (Dret_1036). Pyruvate can then be either activated to phosphoenolpyruvate by the enzyme pyruvate, water dikinase (Dret_0098) to enable gluconeogenesis or is further carboxylated to oxaloacetate by pyruvate carboxylase, which is encoded by two separate genes (Dret_0690 and Dret_1120). Alternatively, pyruvate can be also used for the synthesis of malate by malic enzyme (Dret_0778), which requires NADP^+^ as cofactor. The remaining major precursors for anabolic reactions can then be produced starting from malate by reactions of the reverse TCA cycle, involving the enzymes fumarase (Dret_1068, Dret_1069), fumarate reductase (Dret_1065, Dret_1066,  and Dret_1067), succinyl-CoA synthetase (Dret_0545) and 2-oxoglutarate ferredoxin oxidoreductase (Dret_1400/Dret_1403). The important five carbon precursor 2-oxoglutarate could also be synthesized from citrate by aconitase (Dret_1771) and isocitrate dehydrogenase (Dret_0439). Genes encoding the enzymes ATP-citrate lyase or citrate synthase were not detected in the annotated genome sequence, so that a closing of the TCA cycle is apparently prevented.

### Defense against osmotic and oxidative stress

Cells of *D. retbaense* inhabit saline environments and hence need appropriate protection against low water activity or varying salt concentrations. The accumulation of compatible solutes is a widespread strategy among microorganisms to protect against osmotic stress. In the distantly related moderately halophilic sulfate-reducing bacterium *Desulfovibrio halophilus,* the organic solutes trehalose and glycine betaine were identified as osmoprotectants [30]. In the genome of *D. retbaense* DSM 5692 several genes could be detected that may be involved in the intracellular synthesis or accumulation of the above mentioned compatible solutes. For instance, the organic solute trehalose can be synthesized from UDP-**D**-glucose and alpha-**D**-glucose 6-phosphate by the enzymes trehalose-6-phosphate synthase (Dret_1902) and trehalose-6-phosphatase (Dret_1903). Alternatively, trehalose may be produced from the reserve carbohydrate glycogen, if the enzymes malto-oligosyltrehalose synthase (Dret_0039) and malto-oligosyltrehalose trehalohydrolase (Dret_0037) are expressed. On the other hand, the gene Dret_0035 encodes a trehalose synthase that can transform maltose directly into trehalose and vice versa, so that an excess of trehalose can be converted to glycogen again.

The second osmotic solute in *D. retbaense* appears to be glycine betaine, which can be accumulated in two different ways: The most efficient way in terms of energy represents the uptake from the environment. Glycine betaine is produced by many cyanobacteria or halophilic anoxygenic photosynthetic bacteria and is released continuously in the environment by excretion or cell lysis. The genome of *D. retbaense* DSM 5692 contains two distinct gene clusters (Dret_0768/Dret_0771 and Dret_22771/Dret_22773) that could encode high affinity ABC transporters for the uptake of glycine betaine. Several separate genes encoding periplasmic glycine betaine binding proteins were also found and could have a function in the regulation of genes in response to the presence of glycine betaine in the environment. An alternative route for the accumulation of glycine betaine is based on the uptake of choline. This quaternary amine is an essential component of eukaryotic cell membranes and hence ubiquitous in most environments. Two genes were found that encode putative choline transporters, Dret_1055 and Dret_2376. Following transport to the cytoplasm, choline could then be oxidized to glycine betaine by the enzymes choline dehydrogenase (Dret_0130) and betaine aldehyde dehydrogenase (Dret_0129).

The sensitivity of the obligately anaerobic species *D. retbaense* to oxygen exposure has not been analyzed in detail, but it can be assumed that it is quite moderate as in most other studied sulfate-reducing bacteria [31]. A close inspection of the annotated genome sequence revealed a complex network of antioxidant proteins protecting cells of this species against oxidative stress. Aerobic respiration was identified as one principal mechanism for the detoxification of oxygen in Gram-negative sulfate-reducers [32]. In *D. retbaense* DSM 5692, genes for the two subunits of a cytochrome *bd* quinol oxidase (Dret_0135 and Dret_0136) were identified. This type of oxidase is the most common terminal oxidase among Gram-negative sulfate-reducers and characterized by a high-affinity to oxygen [32,33]. For the detoxification of reactive oxygen species that emerge from the contact of oxygen with cellular redox enzymes several protection systems seem to be present. A di-heme cytochrome *c* peroxidase (Dret_1885) that is probably localized in the periplasmic space [34] is able to reduce H_2_O_2_ to water, whereas a catalase (Dret_1236) produces oxygen from the inactivation of H_2_O_2_. On the other hand, in the cytoplasm multiprotein complexes containing rubredoxins (Dret_0886, Dret_0139), rubrerythrins (Dret_0191, Dret_1205, Dret_1644, Dret_2310) and desulfoferrodoxin (Dret_0140) could establish electron transfer systems for the reduction of superoxide radicals and H_2_O_2_ [35,36]. Finally, cellular proteins and lipids that became damaged by reactive oxygen species could be repaired by a methionine sulfoxide reductase (Dret_2264), peroxiredoxin (Dret_2393) and an alkylhydroperoxidase (Dret_1223).

Thus, based on the results of the genome analysis it seems that this species is very well adapted to frequent changes in salinity and redox conditions in its natural environment, the sediments of hypersaline lakes.
